# SINGLE-SHOT FEMORAL NERVE BLOCK VERSUS LOCAL INFILTRATION ANALGESIA FOR PATIENTS UNDERGOING TOTAL KNEE ARTHROPLASTY: A SYSTEMATIC REVIEW AND META-ANALYSIS

**DOI:** 10.1590/1413-785220263406e309147

**Published:** 2026-11-09

**Authors:** Andre Richard da Silva Oliveira, Rodrigo Bernstein Conde, Elcio Machinski, Vinicius Furtado da Cruz, Camilo Partezani Helito, Andre Giardino Moreira da Silva, Daniel Peixoto Leal

**Affiliations:** 1 Universidade Federal da Paraiba, Joao Pessoa, PB, Brazil.; 2 Fundacion Barcelo, Buenos Aires, Argentina.; 3 Universidade Estadual de Ponta Grossa, Ponta Grossa, PR, Brazil.; 4 Pontificia Universidade Catolica do Parana, Londrina, PR, Brazil.; 5 Universidade de Sao Paulo, Faculdade de Medicina, Hospital das Clinicas (HCFMUSP), Instituto de Ortopedia, Grupo do Joelho, Sao Paulo, SP, Brazil.

**Keywords:** Arthroplasty, Replacement, Knee, Pain Management, Nerve Block, Anesthesia, Local, Artroplastia do Joelho, Manejo da Dor, Bloqueio Nervoso, Anestesia Local

## Abstract

**Objective:**

Early postoperative rehabilitation is essential for optimal outcomes after total knee arthroplasty (TKA). Femoral nerve block and local infiltration analgesia (LIA) are commonly used for postoperative pain control. However, the relative efficacy of single-shot femoral nerve block (sFNB) versus LIA remains uncertain. This meta-analysis compared both strategies in patients undergoing TKA.

**Methods:**

Medline, Cochrane Library, and Embase databases were searched for randomized controlled trials (RCTs) comparing sFNB and LIA in TKA patients. Outcomes included postoperative pain scores, opioid consumption, range of motion (ROM), and length of hospital stay.

**Results:**

Of 922 studies identified, 11 RCTs comprising 1,109 patients were included. No significant differences were observed between groups regarding early postoperative pain scores (mean difference [MD] = 0.25 points; 95% confidence interval [CI]: −0.19 to 0.69) or opioid consumption (MD = 0.55 mg; 95% CI: −3.28 to 4.38). However, the LIA group demonstrated improved ROM on postoperative day 2 (MD = −6.15 degrees; 95% CI: −9.16 to −3.14) and shorter hospital stay (MD = 0.23 days; 95% CI: 0.06 to 0.41).

**Conclusions:**

LIA was associated with improved ROM on the second postoperative day and shorter hospital stay compared with sFNB, without significant differences in pain scores or opioid consumption. *Level of Evidence II; Systematic review and meta-analysis of randomized clinical trials.*

## RESUMO

**Objetivo:**

A reabilitação pós-operatória precoce é essencial para resultados ideais após a artroplastia total do joelho (ATJ). O bloqueio do nervo femoral e a analgesia por infiltração local (AIL) são comumente utilizados para controle da dor pós-operatória. No entanto, a eficácia relativa do bloqueio femoral em dose única (BNFu) versus AIL permanece incerta. Esta meta-análise comparou ambas as estratégias em pacientes submetidos à ATJ.

**Métodos:**

As bases de dados Medline, Cochrane Library e Embase foram pesquisadas em busca de ensaios clínicos randomizados (ECRs) comparando BNFu e AIL em pacientes submetidos à ATJ. Os desfechos incluíram escores de dor pós-operatória, consumo de opioides, amplitude de movimento (ADM) e tempo de internação hospitalar.

**Resultados:**

Dos 922 estudos identificados, 11 ECRs, compreendendo 1.109 pacientes, foram incluídos. Não foram observadas diferenças significativas entre os grupos em relação aos escores de dor pós-operatória precoce (diferença média [DM] = 0,25 pontos; intervalo de confiança [IC] de 95%: −0,19 a 0,69) ou ao consumo de opioides (DM = 0,55 mg; IC 95%: −3,28 a 4,38). No entanto, o grupo AIL demonstrou melhor ADM no segundo dia pós-operatório (DM = −6,15 graus; IC 95%: −9,16 a −3,14) e menor tempo de internação hospitalar (DM = 0,23 dias; IC 95%: 0,06 a 0,41).

**Conclusões:**

A AIL foi associada a melhor ADM no segundo dia pós-operatório e menor tempo de internação hospitalar em comparação ao BNFu, sem diferenças significativas nos escores de dor ou no consumo de opioides. *Nível de evidência II; Revisão sistemática e meta-análise de ensaios clínicos randomizados.*

## INTRODUCTION

The number of total knee arthroplasty (TKA) procedures is expected to rise significantly in the coming years, mainly due to population aging and the increasing prevalence of obesity and osteoarthritis.^1^

Early postoperative rehabilitation in TKA, with progressive gains in range of motion (ROM), pain control, and early ambulation, is a key factor in achieving favorable outcomes.^2-5^ Femoral nerve block (FNB) and local infiltration analgesia (LIA) are among the options for perioperative analgesia in TKA. A femoral nerve block can be administered as a single dose (sFNB) or as a continuous block (cFNB) via a catheter infusion, aiming to extend the analgesic effect of sFNB, which has been reported to last up to 24 hours.^6^ Similarly, LIA has been reported to last 24–48 hours.^7^

Although reduced opioid consumption was observed in the cFNB group, previous meta-analyses comparing sFNB and cFNB did not find significant differences in the visual analog scale (VAS) at 48 hours postoperatively, nor any significant differences in length of hospital stay.^8,9^ Therefore, despite offering benefits over sFNB, the cumulative effect of cFNB may be limited in the context of multimodal analgesic management. Since cFNB may be associated with an increased risk of falls,^10-13^ potentially leading to major and catastrophic complications, sFNB remains a good alternative, especially in patients with other risk factors for falls.

Previous meta-analyses comparing FNB and LIA included both single-shot and continuous forms of FNB and suggested that both FNB and LIA are equally effective in controlling postoperative pain, although LIA is superior in reducing postoperative opioid consumption.^14^ While some randomized clinical trials (RCTs) have shown lower pain scores on the first postoperative day^15-17^ and greater range of motion on the second postoperative day in patients undergoing LIA,^18-20^ others have demonstrated lower opioid consumption within the first 24 hours in patients undergoing single-shot femoral nerve block.^21,22^ Additionally, some studies have failed to show any significant difference between the two methods.^16,18,19,23^ In this context, this meta-analysis of RCTs aims to compare the efficacy of sFNB versus LIA following TKA.

## MATERIALS AND METHODS

This systematic review and meta-analysis was conducted following the guidelines outlined in the Cochrane Collaboration Handbook for Systematic Reviews of Interventions and the Preferred Reporting Items for Systematic Reviews and Meta-Analyses (PRISMA) Statement.^24,25^ The protocol was registered in PROSPERO (CRD42024548788).

### Study Eligibility

The studies included in this meta-analysis met the following criteria: (1) RCTs; (2) comparing sFNB to LIA for the postoperative pain management in patients undergoing TKA. LIA was defined as the use of local anesthetics infiltrated into the skin and soft tissues or the knee joint capsule; sFNB was defined as the single administration of anesthetics around the nerve. Furthermore, studies were only included if they reported any of the clinical outcomes of interest, with adequately reported data, especially continuous outcomes reported as mean and standard deviation; when unavailable, methods recommended by the Cochrane Handbook were applied.^24^ Only peer-reviewed studies were considered, without time or language restrictions.

### Literature Search, Study Selection and Data Abstraction

Medline, the Cochrane Library, and Embase were systematically searched from database inception to May 2024 using the following search terms: “knee arthroplasty”, “local infiltration analgesia”, “femoral nerve block” (the complete search strategy is available in Supplementary Material 1).

After removing duplicates, two authors independently screened titles and abstracts to identify articles that met the eligibility criteria. They then assessed full texts of the selected studies to confirm inclusion. Disagreements were resolved by consulting a third, more experienced reviewer who made the final decision.

Two authors independently extracted data from the included studies using pre-defined criteria, including study characteristics (author, local, year of publication, sample size), mean differences and number of events for outcomes of interest, and participants and intervention details (population attributes, substances, and methods used in the interventions).

### Outcomes

The primary outcome of interest was postoperative pain scores at rest and during mobilization (active or passive) measured on postoperative day 1 (POD1) and postoperative day 2 (POD2), using the VAS for pain or the Numeric Rating Scale (NRS) if VAS was unavailable. Secondary outcomes of interest include (1) opioid consumption measured in milligrams (mg) on POD1 and POD2, (2) ROM measured in degrees on POD1 and POD2, and (3) length of hospital stay measured in days after surgery.

### Data Analysis

The mean differences (MD) for continuous outcomes were pooled with the Generic Inverse-Variance method. Effect estimates were reported with 95% confidence intervals (CI), using a random-effects model with the Restricted Maximum Likelihood approach. Heterogeneity was assessed with I^2^ statistics, with values > 40% considered moderate.

Additionally, sensitivity analyses were performed using the leave-one-out method to investigate the impact of each study on pooled outcomes with heterogeneity greater than 40%. Subgroup analyses were conducted for (1) studies that included epinephrine in the drug cocktail, (2) morphine in the drug cocktail, (3) ketorolac in the drug cocktail.

### Risk of Bias Assessment

The risk of bias in the included studies was assessed using the Cochrane risk-of-bias tool for randomized trials (RoB 2). Each study was evaluated for potential bias across five domains: the randomization process, deviations from intended interventions, missing outcome data, measurement of outcomes, and selection of the reported result. Overall bias was classified as ‘Low,’ ‘Some concerns,’ or ‘High’ risk based on these domains. The publication bias was assessed through funnel plots and Egger's test.

## RESULTS

### Study Selection and Baseline Characteristics

The systematic search yielded 922 results. After removing duplicate records and excluding ineligible studies, 37 studies remained for full eligibility assessment based on the inclusion criteria. A total of 11 RCTs were included in this meta-analysis (Figure 1).^15-23,26,27^

**Figure 1:**
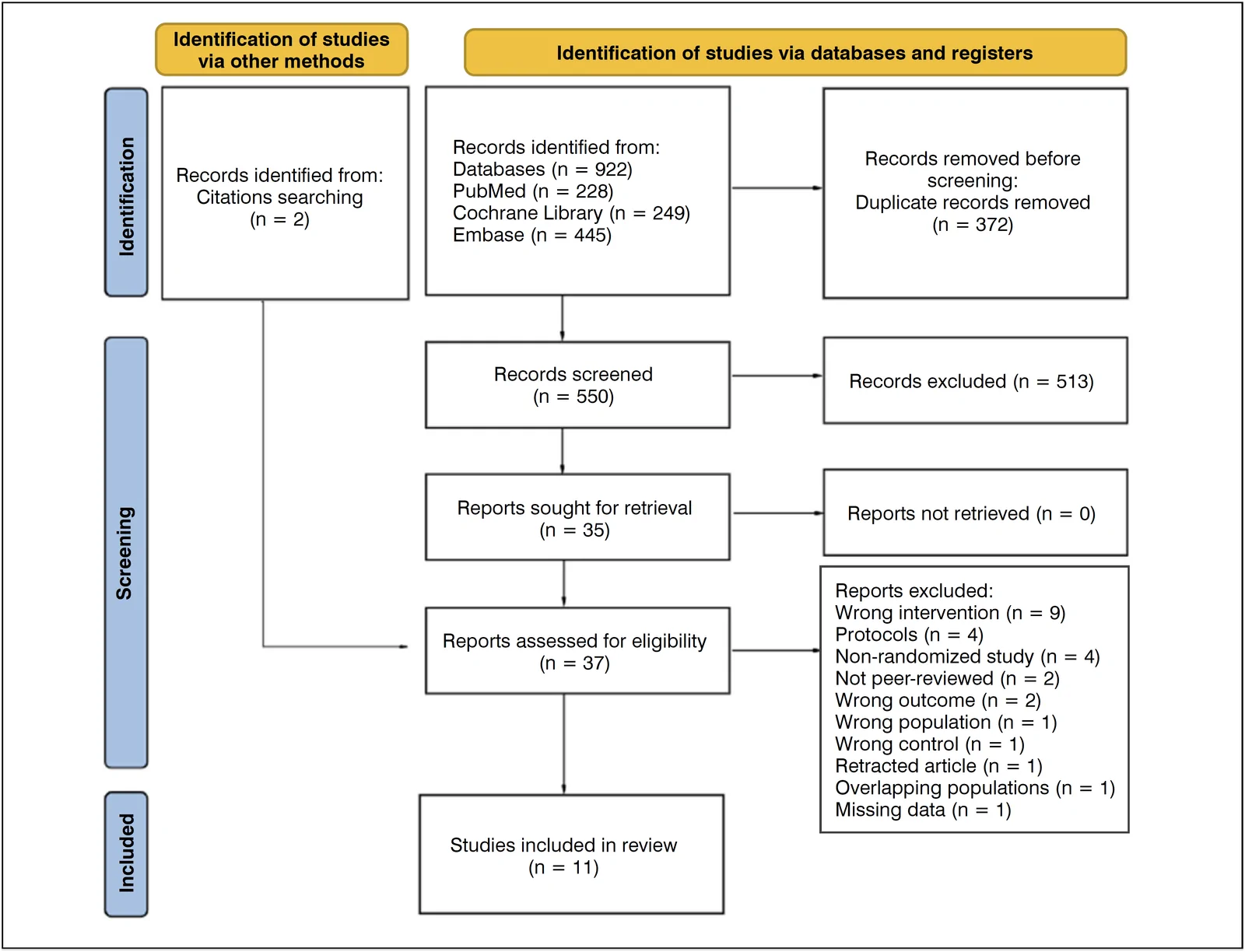
Preferred Reporting Items for Systematic reviews and Meta-Analyses (PRISMA) flow diagram of the study selection.

The meta-analysis included a total of 1,109 patients, with 551 (49.7%) undergoing LIA and 558 (50.3%) receiving sFNB. Baseline characteristics of the studies are summarized in Table 1.

**Table 1:** Baseline characteristics of included studies.

Study	Type of TKA	Local	sFNB† substances	LIA$ substances	Patients sFNB†/LIA$	Anesthesia method	Female, % sFNB/LIA	Age£, y sFNB/LIA	Body Mass Index£, kg/m^2^ sFNB/LIA
Ashraf et al., 2013^15^	Primary	England	30 mL of 0.2% ropivacaine	150 mL 0.2% ropivacaine, 1 mL 1:1000 adrenaline, 30 mg ketorolac	21/19	Spinal	NA	NA	NA
Chandrashekaraiah et al., 2021^21^	Primary	Bahrain	25mL 0.5% bupivacaine (125 mgs)	50 mL of 0.25% bupivacaine (125 mgs), 10 mg of morphine (1mL) and 99 mL of normal saline	29/30	Spinal	65.5/66.6	65.31/61.70	NA
Choi et al., 2016^16^	Primary	Canada	Ropivacaine 0.5% 20 mL	Ropivacaine 0.2% with epinephrine 10 μg/mL, 150 mL and ketorolac 30 mg	39/41	Spinal	49/51	65.2/65.9	31.4/29.3
Craik et al., 2022^26^	Primary	Australia	20 mL 0.2% ropivacaine	200 mL of 0.2% ropivacaine + 5 mL of 0.2% ropivacaine per hour for 24 hours	14/13	Spinal	43/54	66/69	31/31
Fan et al., 2016^23^	Primary	China	20mL of ropivacaine 0.5%	50 mL of cocktail mixture containing morphine (1 mL: 10 mg), ropivacaine (10 mL: 100 mg), and diprospan (1 mL: 5 mg betamethasone dipropionate and 2 mg betamethasone sodium phosphate)	78/79	General	79.5/86	68.36/67.59	21.3/21.6
Ismail et al., 2020^17^	NA	Egypt	30 mL of bupivacaine 0.25% with adrenaline 1:200,000	200 mg 0.5% bupivacaine, 5 mg morphine and 200 μg epinephrine mixed with sterile normal saline solution to make up a combined volume of 50 mL	65/65	General	53.8/53.8	62.88/62.97	27.62/27.4
Lopes Júnior et al., 2024^18^	Primary	Brazil	150 mg of ropivacaine and 150 μg of clonidine	150 μg of clonidine, 30 mg of ketorolac, 375 mg of ropivacaine, and 1 mg of epinephrine diluted in 50 mL of saline solution	50/50	Spinal	78/76	68.1/67.7	NA
Moghtadaei et al., 2014^27^	Primary	Iran	20 mL of ropivacaine 10 mg/mL was injected	150 mL: 300 mg ropivacaine 1%, 30 mg ketorolac and 0.5 mg 1:100,000 epinephrine	18/18	Spinal	33.3/27.8	67.4/64	28/27
Ng et al., 2020^22^	Primary	England	20 mL 0.375% levobupivacaine	40 mL 0.25% bupivacaine with adrenaline 1:200,000, diluted with 110 mL of 0.9% saline, making a total volume of 150 mL	79/69	Spinal	49.3/44.9	NA	NA
Wall et al., 2017^19^	Primary	England	75 mg of 0.25% levobupivacaine hydrochloride	150 mg of 0.25% levobupivacaine hydrochloride, 10 mg morphine sulfate, 30 mg ketorolac trometamol and 0.25 mg of adrenaline, all diluted with 0.9% saline to make a volume of 150 ml	131/131	Spinal or general	61.1/58.8	68.2/68.7	NA
Ye et al., 2020^20^	Primary	China	0.25% ropivacaine 20 mL (89.4 mg)	1% ropivacaine hydrochloride 447 mg, flurbiprofen ester 100 mg, adrenal 1 mg, and dexamethasone 5 mg were mixed in saline. A 100 mL cocktail was prepared	35/35	General	68.5/65.7	64.5/65.5	25/23.7

Captions:

† Single Femoral Nerve Block

$ Local infiltration analgesia

£ Mean.

### Pain scores at rest

Pain scores at rest on POD1 showed no significant difference between sFNB and LIA (MD = 0.25 points; 95% CI: −0.19 to 0.69; p = 0.27; I^2^ = 61%; Figure 2). This analysis included eight RCTs with 944 patients. The same pattern was observed on POD2 (MD = 0.08; 95% CI: −0.30 to 0.46; p = 0.68; I^2^ = 41%; Supplementary Figure 1).

**Figure 2:**
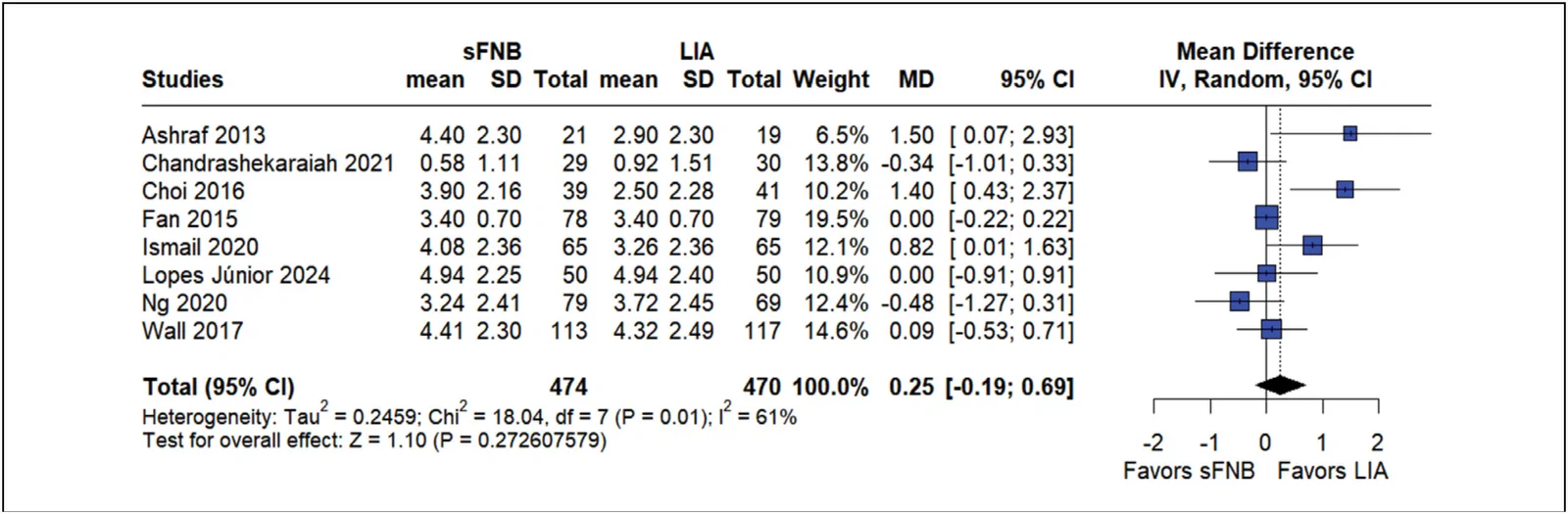
Pain scores at rest on postoperative day 1.

### Pain scores during mobilization

The analysis of pain scores during mobilization also showed no significant differences between groups on POD1 (656 patients) (MD = 0.34; 95% CI: −0.66 to 1.34; p = 0.50; I^2^ = 81%; Figure 3) and on POD2 (467 patients) (MD = −0.04; 95% CI: −0.28 to 0.20; p = 0.75; I^2^ = 23%; Supplementary Figure 2).

**Figure 3:**
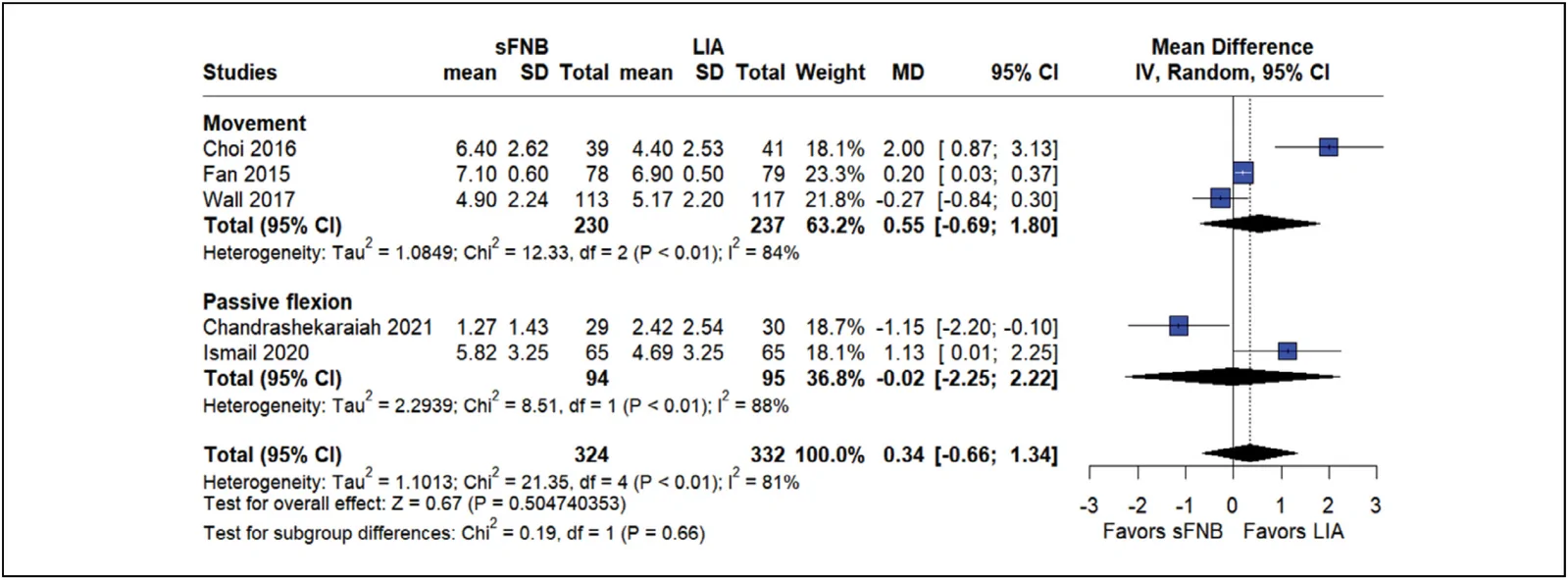
Pain scores during mobilization on postoperative day 1.

### Opioid consumption

Eight RCTs with 955 patients evaluated opioid consumption 24 hours after surgery (MD = 0.55 mg; 95% CI: −3.28 to 4.38; p = 0.77; I^2^ = 85%; Figure 4). For opioid consumption on POD2, results remained non-significant (MD = −0.32 mg; 95% CI: −1.00 to 0.35; p = 0.35; I^2^ = 8%; Supplementary Figure 3).

**Figure 4:**
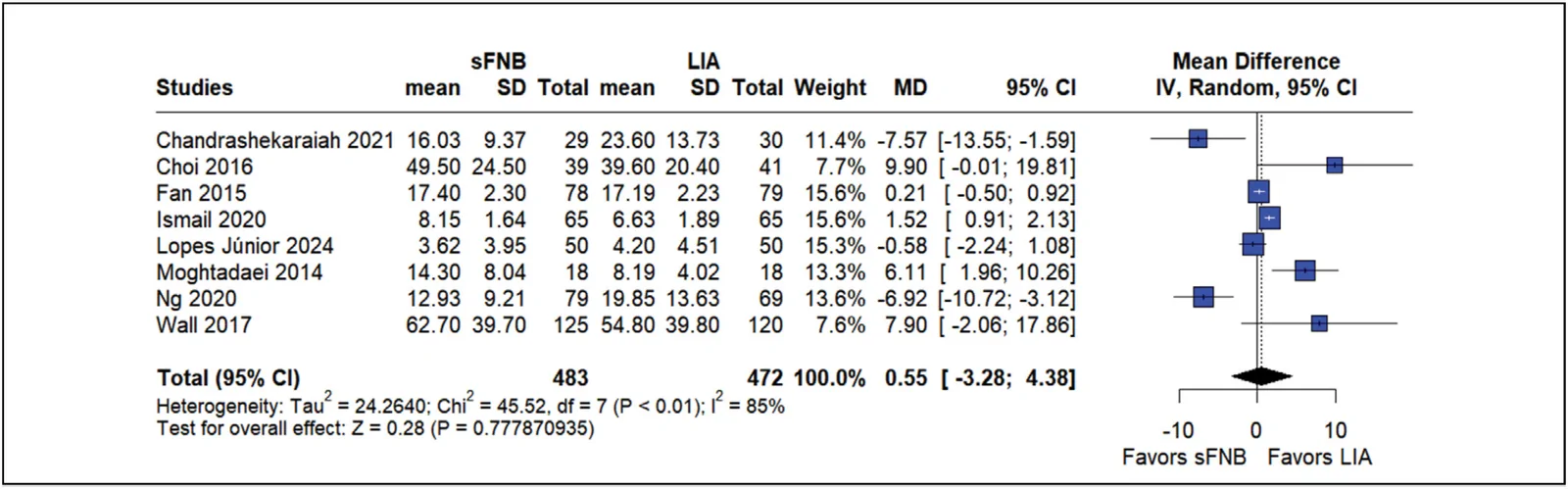
Opioid consumption on postoperative day 1.

### Range of motion

Three RCTs with 405 patients assessed ROM on POD1, showing no significant difference between the groups (MD = −4.53 degrees; 95% CI: −10.11 to 1.05; p = 0.11; I^2^ = 0%; Figure 5). For ROM on POD2, the analysis indicated a wider ROM in the LIA group (MD = −6.15 degrees; 95% CI: −9.16 to −3.14; p < 0.01; I^2^ = 0%; Figure 6).

**Figure 5:**
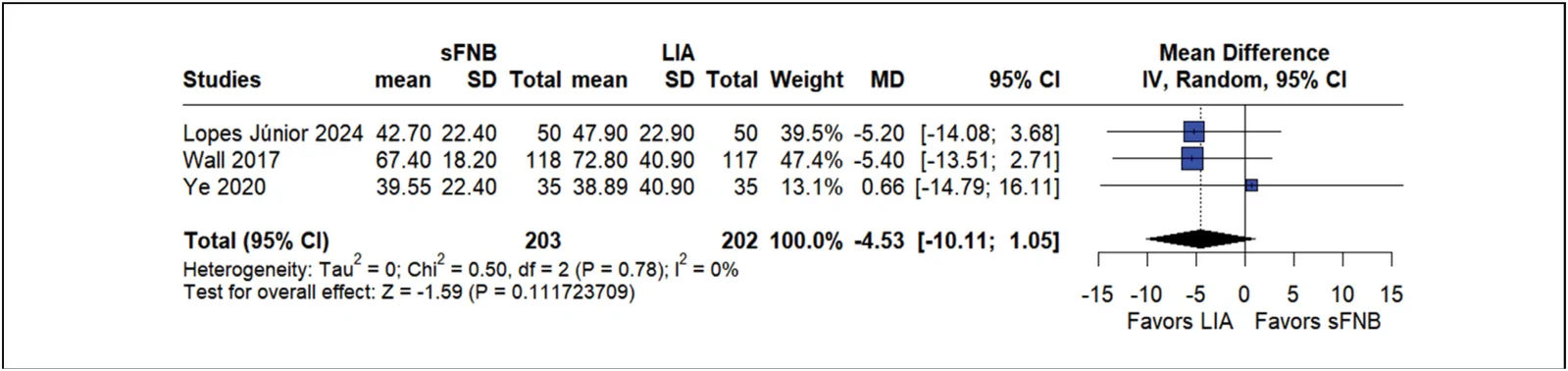
Range of motion on postoperative day 1.

**Figure 6:**
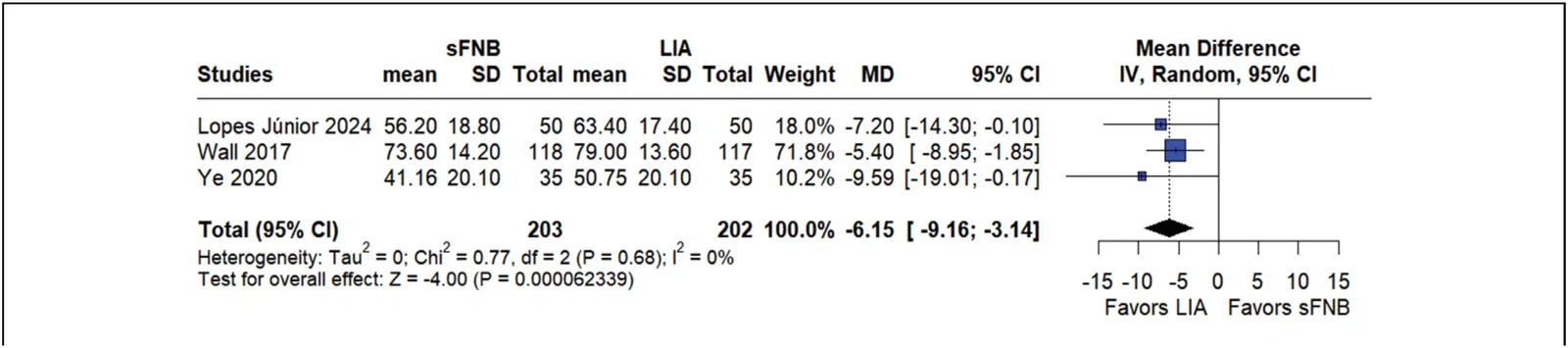
Range of motion on postoperative day 2.

### Length of hospital stay

Four RCTs with 377 patients evaluated the length of hospital stay, finding that the LIA group had a shorter duration of hospitalization (MD = 0.23 days; 95% CI: 0.06 to 0.41; p < 0.01; I^2^ = 0%; Figure 7).

**Figure 7:**
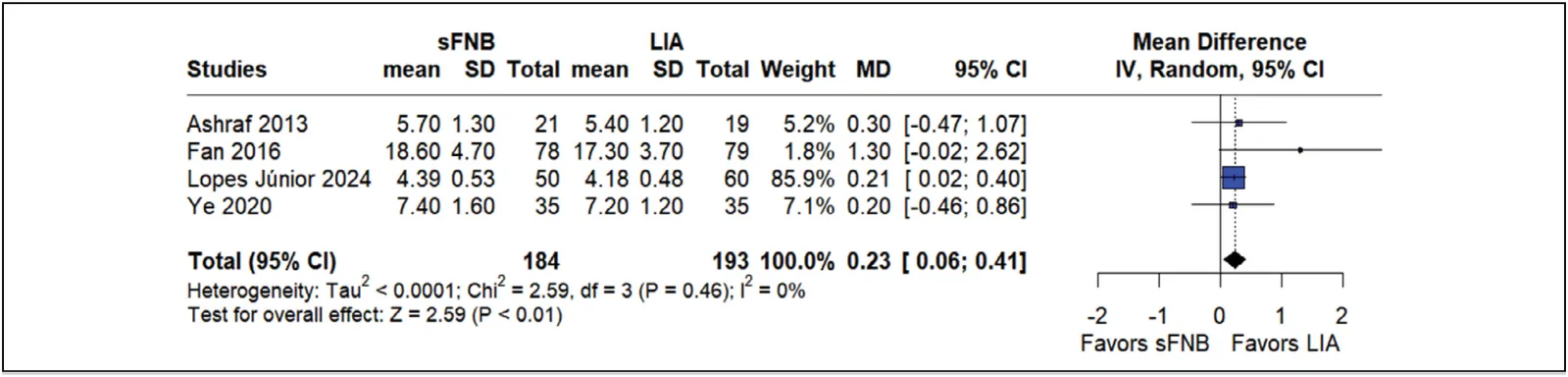
Length of hospital stay.

### Subgroup Analysis

Subgroup analyses according to the use of epinephrine, morphine, or ketorolac in the LIA cocktail showed no significant differences between sFNB and LIA regarding postoperative pain scores on POD1 or opioid consumption on POD1 and POD2 (Supplementary Figures 4–12).

### Sensitivity Analysis

The leave-one-out analyses for postoperative pain score at rest on POD1 (Supplementary Figure 13) and opioid consumption on POD1 (Supplementary Figure 14) showed a trend consistent with the overall results, with no single study identified as having a predominant impact on heterogeneity.

The leave-one-out analysis for pain scores at rest on POD2 showed that omission of Fan et al^23^ reduced the heterogeneity to unimportant, while the results remained statistically non-significant (I^2^ decreased from 41% to 0%; MD = 0.33; 95% CI: −0.10 to 0.77; Supplementary Figure 15).

### Quality Assessment

The quality assessment using the RoB2 tool indicated that none of the studies had an overall high risk of bias. Most studies were classified as having some concerns, while four studies^16,17,19,23^ were rated as having a low overall risk of bias (Supplementary Figure 16).

A funnel plot was used to assess potential publication bias for pain scores on POD1, revealing symmetry (Supplementary Figure 17), suggesting no evidence of publication bias. Additionally, an Egger's test confirmed the absence of asymmetry (t = 1.284; p = 0.246).

## DISCUSSION

This systematic review and meta-analysis specifically compared sFNB with LIA for postoperative pain management following TKA. Previous meta-analyses included both continuous and single-shot FNBs, potentially confounding the comparative outcomes^14,28-31^. The present study included 11 RCTs with a total of 1,109 patients. The main findings were: (1) there were no statistically significant differences between the two groups in terms of postoperative pain scores or opioid consumption; (2) the LIA group demonstrated a shorter hospital stay and improved range of motion on postoperative day 2; and (3) subgroup analyses involving LIA cocktails containing ketorolac, morphine, or epinephrine showed a general trend toward non-significance in pain control.

For pain at rest on POD1, our findings are consistent with those of previous meta-analyses which included both sFNB and cFNB in the analysis.^14,28-31^ Additionally, three of them evaluated pain at rest on POD2 and also reported no significant differences between the two modalities.^28,30,31^ Four of the previous meta-analyses assessed pain during mobilization,^14,28,29,31^ but only the study by Mei et al.^29^ found differences in pain scales, with the FNB group presenting lower pain scores on POD1. In neither the previous meta-analyses nor our meta-analysis—which included only sFNB—did the mean differences in pain scale values reach the minimal clinically important difference for TKA, defined as 2.26 centimeters for improvement and 2.91 centimeters for worsening on the visual analog scale.^32^

The present study found no significant differences between sFNB and LIA regarding opioid consumption. Previous meta-analyses also found no significant differences in opioid consumption on POD1 and POD2,^28,29,31^ nor in total opioid morphine equivalent consumption throughout the entire hospital stay.^29,30^ Conversely, the meta-analysis by Liu et al.^14^ found that LIA was associated with lower opioid consumption on POD1. Overall, the analgesic efficacy of sFNB and LIA seems to be comparable, and possible differences in terms of analgesia may represent a minor effect in the role of multimodal analgesia. The combination of both peripheral blocks and LIA methods is generally suggested to enhance postoperative analgesia.^33^

A concern with the use of FNB is the occurrence of quadriceps motor block, which could increase the risk of postoperative falls, although this remains controversial in the literature.^11-13,34-37^ A study by Memtsoudis et al.^34^ with a large database involving 400 hospitals and 191,570 patients in the United States found no association between inpatient falls and peripheral nerve block (PNB). Conversely, studies evaluating risk factors for falls following TKA have identified advanced age, obesity, and the use of cFNB as being associated with an increased risk of falls.^11-13,37^ Proprioception, ROM, and quadriceps strength were also associated with the risk of falling.^35,36^ Regarding ROM, the present pooled analysis found no significant difference between the analgesic methods on POD1, but observed a 6.15-degree greater ROM in the LIA group on POD2. Our analysis included the studies by Lopes Júnior et al.,^18^ Wall et al.,^19^ and Ye et al.,^20^ which all reported greater ROM in the LIA group, with mean differences of 7.2º, 5.4º, and 9.59º, respectively. As the reported duration of analgesic effect for LIA^7^ is slightly longer than that of sFNB,^6^ a possible explanation for the improved ROM on POD2 is the prolonged pain relief provided by LIA. Matsumoto et al.^36^ investigated the correlation between falls and ROM in TKA patients between 6 and 12 months postoperatively and found that reduced postoperative knee flexion and ankle plantarflexion were factors related to falls, with patients who experienced falls demonstrating a mean knee range of motion 9.3 degrees lower than non-fallers. Among the studies included in the present meta-analysis, Choi et al.^16^ reported no falls, Ng et al.^22^ reported one fall in each group, and Lopes Júnior et al.^18^ reported one fall in the sFNB group without further consequences. Due to the limited number of reported events, a pooled analysis regarding the risk of falling could not be performed.

Reducing the length of hospital stay is beneficial for minimizing clinical complications, reducing the risk of prosthesis-related infections, and lowering healthcare costs.^38-40^ In the present study, the LIA group had a 0.23-day shorter hospital stay than the sFNB group, equivalent to approximately 6 hours. Similarly, Albrecht et al.^28^ reported a 0.3-day shorter length of stay in the LIA group. Reported lengths of hospital stay after TKA procedures vary widely in the literature,^40-45^ due to differences in healthcare systems, institutional protocols, hospital infrastructure, and patient-related factors such as age, smoking status, baseline comorbidities, preoperative corticosteroid use, and the American Society of Anesthesiologists (ASA) Physical Status classification.^41,42^ El Bitar et al.^41^ analyzed data from 1,924,432 patients in the Nationwide Inpatient Sample database in the United States and found that 74.8% of those who underwent TKA had a hospital stay of three days or less. Among the studies included, Fan et al.^23^ reported substantially longer hospital stays than the other studies, representing an outlier within the dataset.

This study has some limitations. The primary limitation is the high heterogeneity observed in opioid consumption and postoperative pain score outcomes. To address this, we have performed subgroup analyses with studies using epinephrine, ketorolac, and morphine in the LIA cocktail. However, the subgroup results remained consistent with the overall findings, and no reduction in heterogeneity was observed, suggesting that adding these substances to LIA does not confer superiority over sFNB or significantly influence heterogeneity. Therefore, heterogeneity may partially stem from variations in analgesia and rehabilitation protocols across studies, including different NSAIDs and analgesics, administration methods, such as patient-controlled devices and fixed opioid doses based on VAS levels. Additionally, leave-one-out analyses were performed, but heterogeneity remained high, and no single study contributed significantly to the heterogeneity in opioid consumption and postoperative pain scores.

## CONCLUSION

This systematic review and meta-analysis comparing local infiltration analgesia (LIA) to single-shot femoral nerve block (sFNB) for postoperative management in TKA patients indicates a 0.23-day shorter hospital stay and greater range of motion on postoperative day two in the LIA group. No statistically significant differences were observed between the two methods regarding opioid consumption or postoperative pain scores.

## Data Availability

The contents are already available.

## References

[1] Klug A, Gramlich Y, Rudert M, Drees P, Hoffmann R, Weißenberger M (2021). The projected volume of primary and revision total knee arthroplasty will place an immense burden on future health care systems over the next 30 years. Knee Surg Sports Traumatol Arthrosc.

[2] Chandrasekaran S, Ariaretnam SK, Tsung J, Dickison D (2009). Early mobilization after total knee replacement reduces the incidence of deep venous thrombosis. ANZ J Surg.

[3] Hannon CP, Fillingham YA, Hamilton WG, Della Valle CJ (2022). Multimodal Analgesia and Anesthesia: Enabling Safe and Rapid Recovery for Total Joint Arthroplasty Patients. J Arthroplasty.

[4] Lei YT, Xie JW, Huang Q, Huang W, Pei FX (2021). Benefits of early ambulation within 24 h after total knee arthroplasty: a multicenter retrospective cohort study in China. Mil Med Res.

[5] Malviya A, Martin K, Harper I, Muller SD, Emmerson KP, Partington PF (2011). Enhanced recovery program for hip and knee replacement reduces death rate. Acta Orthop.

[6] Szczukowski MJ, Hines JA, Snell JA, Sisca TS (2004). Femoral nerve block for total knee arthroplasty patients: a method to control postoperative pain. J Arthroplasty.

[7] Seangleulur A, Vanasbodeekul P, Prapaitrakool S, Worathongchai S, Anothaisintawee T, McEvoy M (2016). The efficacy of local infiltration analgesia in the early postoperative period after total knee arthroplasty: A systematic review and meta-analysis. Eur J Anaesthesiol.

[8] Li S, Zhou J, Li X, Teng X, Li Y, Du C (2020). Analgesic Impact of Single-Shot Versus Continuous Femoral Nerve Block After Total Knee Arthroplasty: A Systematic Review and Meta-Analysis. Adv Ther.

[9] Ma HH, Chou TFA, Tsai SW, Chen CF, Wu PK, Chen WM (2020). The efficacy of continuous versus single-injection femoral nerve block in Total knee Arthroplasty: a systematic review and meta-analysis. BMC Musculoskelet Disord.

[10] Ilfeld BM (2014). Single-injection and continuous femoral nerve blocks are associated with different risks of falling. Anesthesiology.

[11] Ilfeld BM, Duke KB, Donohue MC (2010). The Association Between Lower Extremity Continuous Peripheral Nerve Blocks and Patient Falls After Knee and Hip Arthroplasty. Anesth Analg.

[12] Wasserstein D, Farlinger C, Brull R, Mahomed N, Gandhi R (2013). Advanced age, obesity and continuous femoral nerve blockade are independent risk factors for inpatient falls after primary total knee arthroplasty. J Arthroplasty.

[13] Johnson RL, Kopp SL, Hebl JR, Erwin PJ, Mantilla CB (2013). Falls and major orthopaedic surgery with peripheral nerve blockade: a systematic review and meta-analysis. Br J Anaesth.

[14] Liu Q, Wang A, Zhang J (2022). The effects of local infiltration anesthesia and femoral nerve block analgesia after total knee arthroplasty: a systematic review and meta-analysis. Ann Transl Med.

[15] Ashraf A, Raut V, Canty S, McLauchlan G (2013). Pain control after primary total knee replacement. A prospective randomised controlled trial of local infiltration versus single shot femoral nerve block. Knee.

[16] Choi S, O'Hare T, Gollish J, Paul J, Kreder H, Thorpe K (2016). Optimizing Pain and Rehabilitation After Knee Arthroplasty: a Two-Center, Randomized Trial. Anesth Analg.

[17] Ismail TI, Elshafie MH (2020). Patient-reported outcomes after femoral nerve block versus periarticular injections in patients undergoing total knee arthroplasty: A randomized controlled study. Indian J Clin Anaesth.

[18] Lopes O, Viana J, Carvalho J, Folle B, Kuhn V, Saggin P (2024). Infiltração periarticular comparada ao bloqueio do nervo femoral único na artroplastia total de joelho: Um estudo prospectivo randomizado. Rev Bras Ortop (Sao Paulo).

[19] Wall P, Parsons N, Parsons H, Achten J, Balasubramanian S, Thompson P (2017). A pragmatic randomised controlled trial comparing the efficacy of a femoral nerve block and periarticular infiltration for early pain relief following total knee arthroplasty. Bone Joint J.

[20] Zhang Y, Feng S, Zhang Y, Sun J, Shi S, Chen X (2020). Early analgesic effect of cocktail periarticular injection versus femoral nerve block in total knee arthroplasty: a single-center randomized double-blind control. Chin J Tissue Eng Res.

[21] Chandrashekaraiah MM, Shah VH, Sahitya PV, Pandey VC, Butt AJ, Mohammed S (2021). Impact of Intra-Articular Local Anesthesia Infiltration versus Femoral Nerve Block for Postoperative Pain Management in Total Knee Arthroplasty. Anesth Essays Res.

[22] Ng YM, Martin F, Waterson HB, Green A, Preece J, Robinson N (2020). A Randomised Controlled Trial of Local Infiltration Analgesia Versus Femoral Nerve Block for Postoperative Analgesia Following Total Knee Arthroplasty. Cureus.

[23] Fan L, Yu X, Zan P, Liu J, Ji T, Li G (2016). Comparison of Local Infiltration Analgesia With Femoral Nerve Block for Total Knee Arthroplasty: a Prospective, Randomized Clinical Trial. J Arthroplasty.

[24] Higgins JPT, Thomas J, Chandler J, Cumpston M, Li T, Page MJ (2023). Cochrane Handbook for Systematic Reviews of Interventions.

[25] Page MJ, McKenzie JE, Bossuyt PM, Boutron I, Hoffmann TC, Mulrow CD (2021). The PRISMA 2020 statement: an updated guideline for reporting systematic reviews. BMJ.

[26] Craik JD, Koerber JG, Krishnan J, Wilson C (2022). Analgesia for Total Knee Arthroplasty: A Randomized Controlled Study Comparing Local Infusion Analgesia, Femoral Nerve Block and Intrathecal Morphine. Open Access J Biomed Sci.

[27] Moghtadaei M, Farahini H, Faiz S, Mokarami F, Safari S (2014). Pain Management for Total Knee Arthroplasty: single-Injection Femoral Nerve Block versus Local Infiltration Analgesia. Iran Red Crescent Med J.

[28] Albrecht E, Guyen O, Jacot-Guillarmod A, Kirkham KR (2016). The analgesic efficacy of local infiltration analgesia vs femoral nerve block after total knee arthroplasty: A systematic review and meta-analysis. Br J Anaesth.

[29] Mei S, Jin S, Chen Z, Ding X, Zhao X, Li Q (2015). Analgesia for total knee arthroplasty: a meta-analysis comparing local infiltration and femoral nerve block. Clinics (Sao Paulo).

[30] Zhang LK, Ma JX, Kuang MJ, Ma XL (2018). Comparison of Periarticular Local Infiltration Analgesia With Femoral Nerve Block for Total Knee Arthroplasty: a Meta-Analysis of Randomized Controlled Trials. J Arthroplasty.

[31] Yun XD, Yin XL, Jiang J, Teng YJ, Dong HT, An LP (2015). Local infiltration analgesia versus femoral nerve block in total knee arthroplasty: A meta-analysis. Orthop Traumatol Surg Res.

[32] Danoff JR, Goel R, Sutton R, Maltenfort MG, Austin MS (2018). How Much Pain Is Significant? Defining the Minimal Clinically Important Difference for the Visual Analog Scale for Pain After Total Joint Arthroplasty. J Arthroplasty.

[33] Aso K, Izumi M, Sugimura N, Okanoue Y, Kamimoto Y, Yokoyama M (2019). Additional benefit of local infiltration of analgesia to femoral nerve block in total knee arthroplasty: double-blind randomized control study. Knee Surg Sports Traumatol Arthrosc.

[34] Memtsoudis SG, Danninger T, Rasul R, Poeran J, Gerner P, Stundner O (2014). Inpatient falls after total knee arthroplasty: the role of anesthesia type and peripheral nerve blocks. Anesthesiology.

[35] Levinger P, Menz HB, Wee E, Feller JA, Bartlett JR, Bergman NR (2011). Physiological risk factors for falls in people with knee osteoarthritis before and early after knee replacement surgery. Knee Surg Sports Traumatol Arthrosc.

[36] Matsumoto H, Okuno M, Nakamura T, Yamamoto K, Hagino H (2012). Fall incidence and risk factors in patients after total knee arthroplasty. Arch Orthop Trauma Surg.

[37] Sharma S, Iorio R, Specht LM, Davies-Lepie S, Healy WL (2010). Complications of Femoral Nerve Block for Total Knee Arthroplasty. Clin Orthop Relat Res.

[38] DeMik DE, Carender CN, An Q, Callaghan JJ, Brown TS, Bedard NA (2022). Longer Length of Stay Is Associated With More Early Complications After Total Knee Arthroplasty. Iowa Orthop J.

[39] Lovald ST, Ong KL, Malkani AL, Lau EC, Schmier JK, Kurtz SM (2014). Complications, mortality, and costs for outpatient and short-stay total knee arthroplasty patients in comparison to standard-stay patients. J Arthroplasty.

[40] Molloy IB, Martin BI, Moschetti WE, Jevsevar DS (2017). Effects of the Length of Stay on the Cost of Total Knee and Total Hip Arthroplasty from 2002 to 2013. J Bone Joint Surg Am.

[41] El Bitar YF, Illingworth KD, Scaife SL, Horberg JV, Saleh KJ (2015). Hospital Length of Stay following Primary Total Knee Arthroplasty: Data from the Nationwide Inpatient Sample Database. J Arthroplasty.

[42] Hart A, Bergeron SG, Epure L, Huk O, Zukor D, Antoniou J (2015). Comparison of US and Canadian Perioperative Outcomes and Hospital Efficiency After Total Hip and Knee Arthroplasty. JAMA Surg.

[43] Burn E, Edwards CJ, Murray DW, Silman A, Cooper C, Arden NK (2018). Trends and determinants of length of stay and hospital reimbursement following knee and hip replacement: evidence from linked primary care and NHS hospital records from 1997 to 2014. BMJ Open.

[44] Styron JF, Koroukian SM, Klika AK, Barsoum WK (2011). Patient vs provider characteristics impacting hospital lengths of stay after total knee or hip arthroplasty. J Arthroplasty.

[45] Long H, Zeng C, Shi Y, Wang H, Xie D, Lei G (2023). Length of stay and inpatient charges of total knee arthroplasty in China: analysis of a national database. Chin Med J (Engl).

