# Supplementary material for: SINGLE-SHOT FEMORAL NERVE BLOCK VERSUS LOCAL INFILTRATION ANALGESIA FOR PATIENTS UNDERGOING TOTAL KNEE ARTHROPLASTY: A SYSTEMATIC REVIEW AND META-ANALYSIS
Source: Acta Ortop Bras. 2026 Nov 9;34(6):e309147. doi: 10.1590/1413-785220263406e309147 (PMC13749637; doi:10.1590/1413-785220263406e309147)
Supplement: Supplementary file 1 [file 1809-4406-aob-34-6-e309147-Suppl01.pdf]

SUPPLEMENTARY MATERIAL

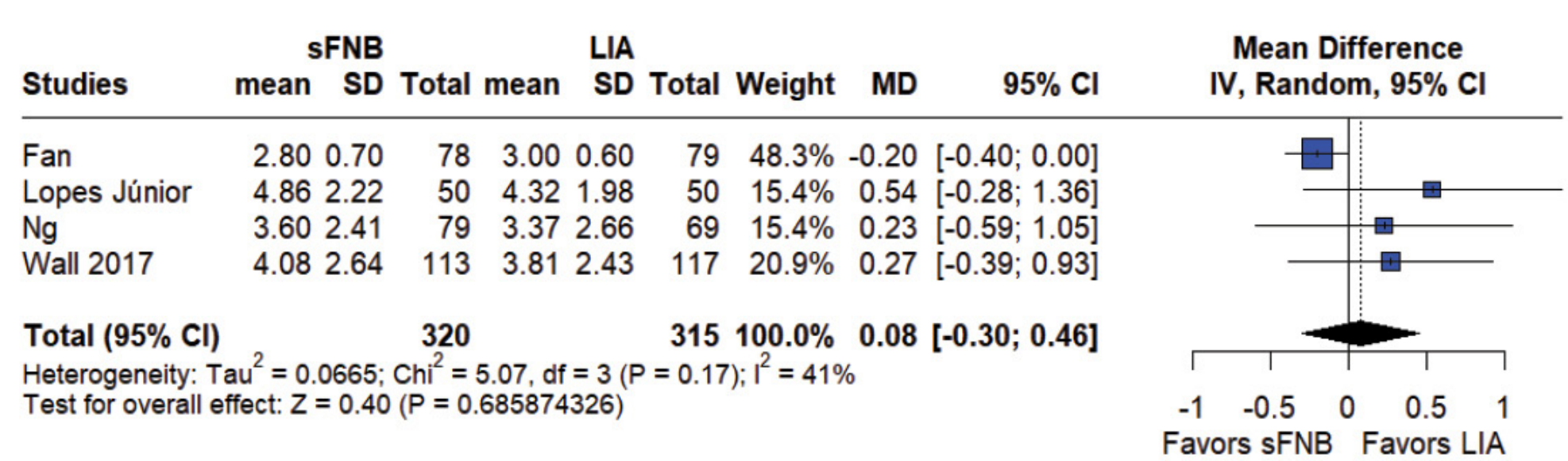

Supplementary Figure 1. Pain scores at rest on postoperative day 2.

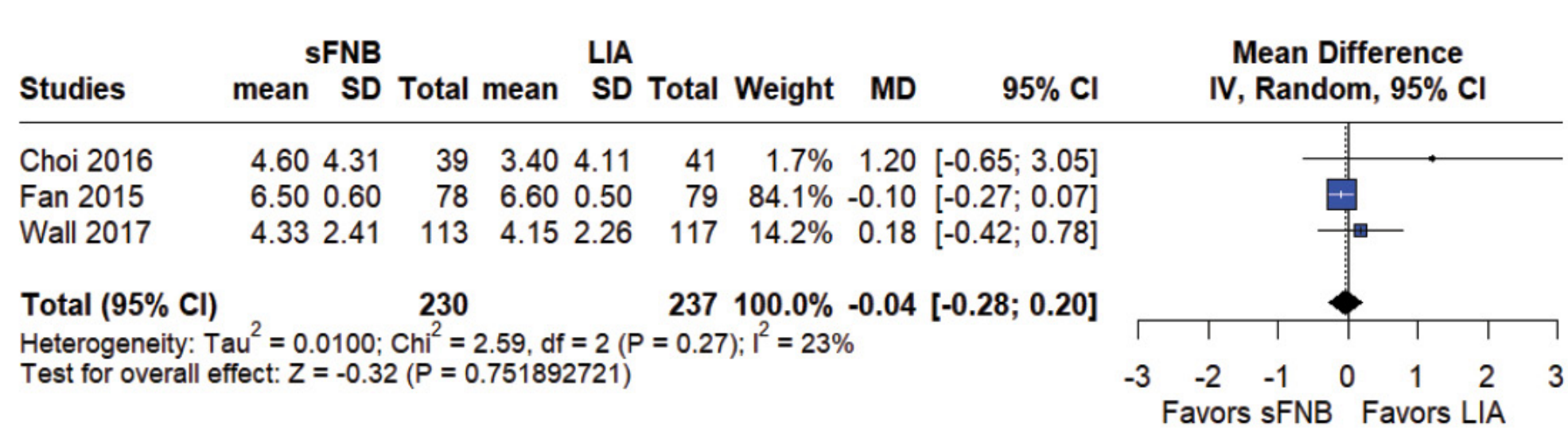

Supplementary Figure 2. Pain scores during mobilization on postoperative day 2.

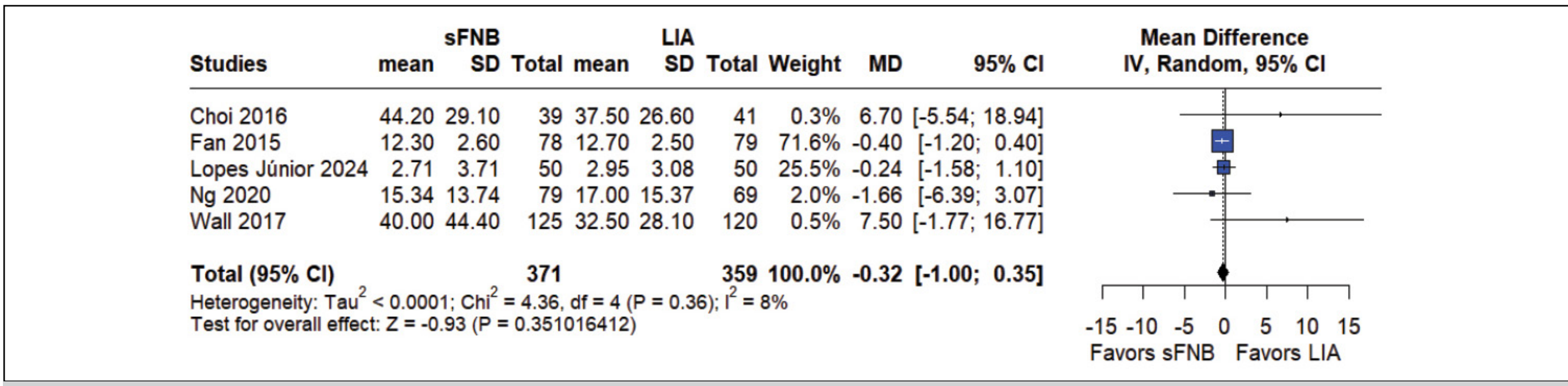

Supplementary Figure 3. Opioid consumption on postoperative day 2.

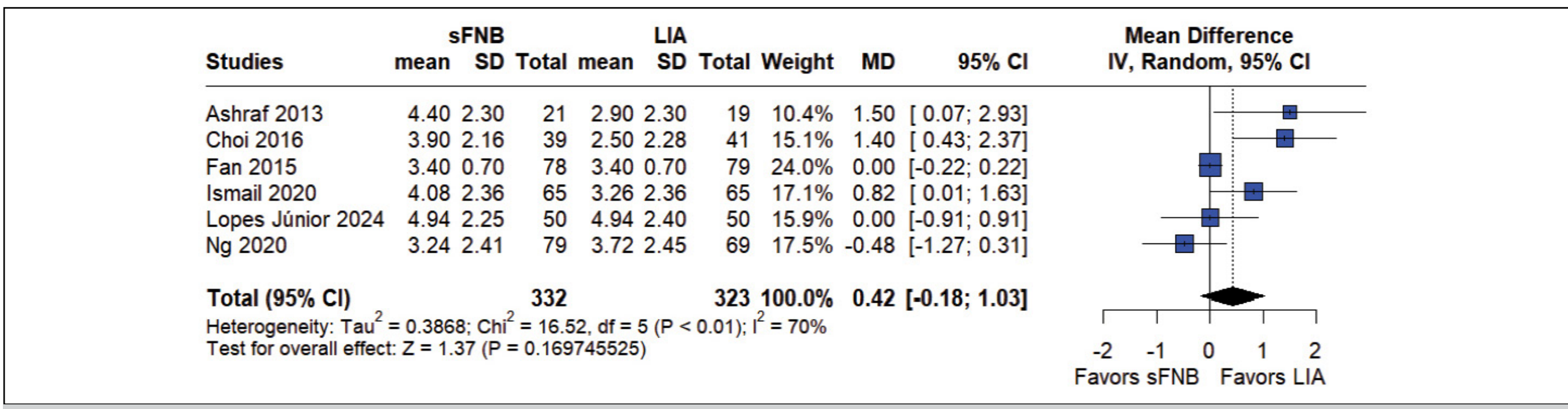

Supplementary Figure 4. Subgroup of studies that used epinephrine in the local infiltration analgesia cocktail for the outcome of pain scores on postoperative day 1.

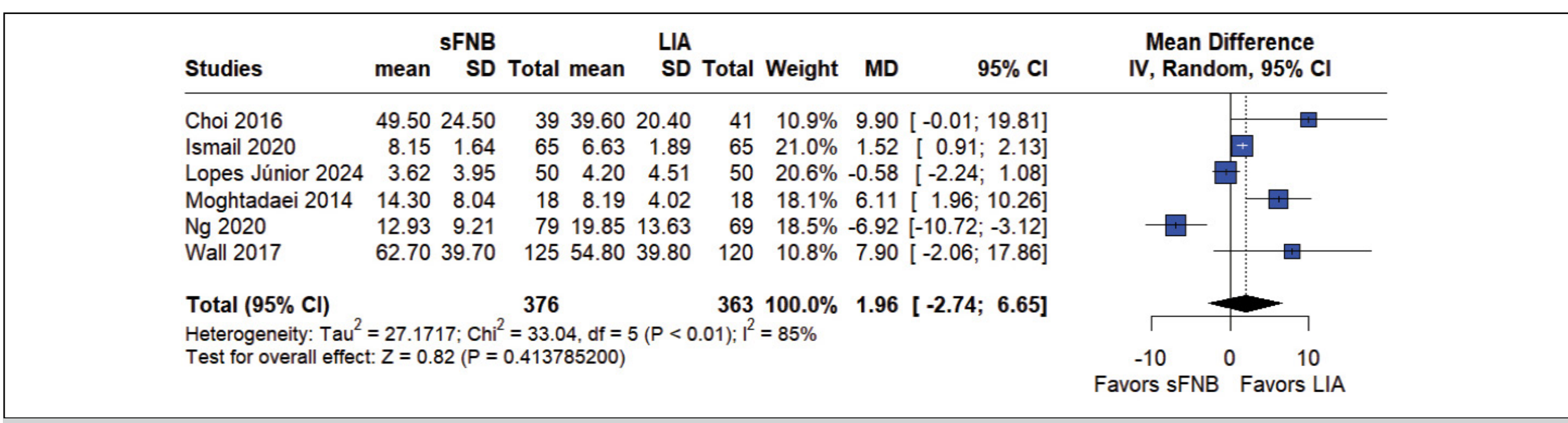

Supplementary Figure 5. Subgroup of studies that used epinephrine in the local infiltration analgesia cocktail for the outcome of opioid consumption on postoperative day 1.

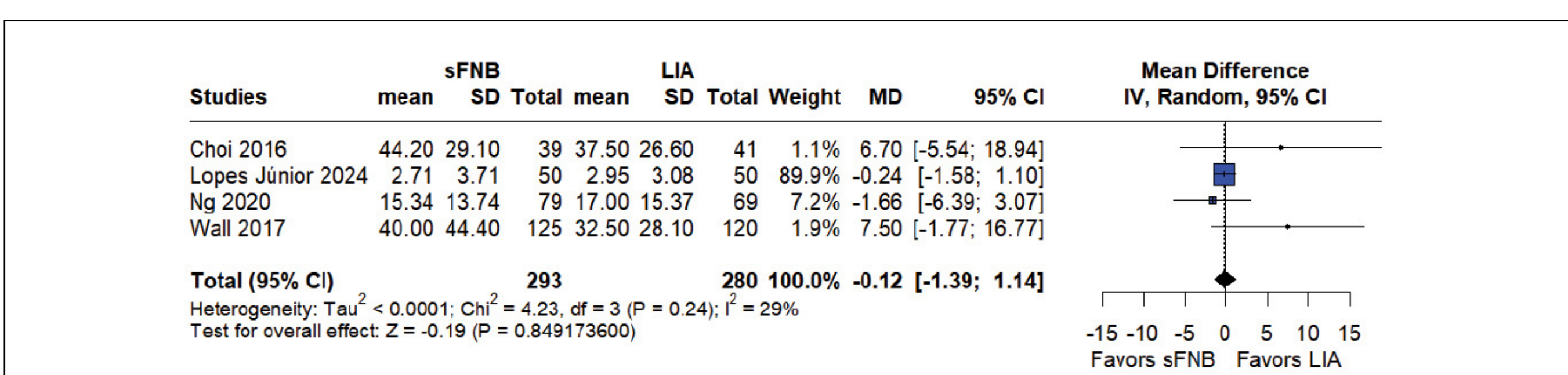

Supplementary Figure 6. Subgroup of studies that used epinephrine in the local infiltration analgesia cocktail for the outcome of opioid consumption on postoperative day 2

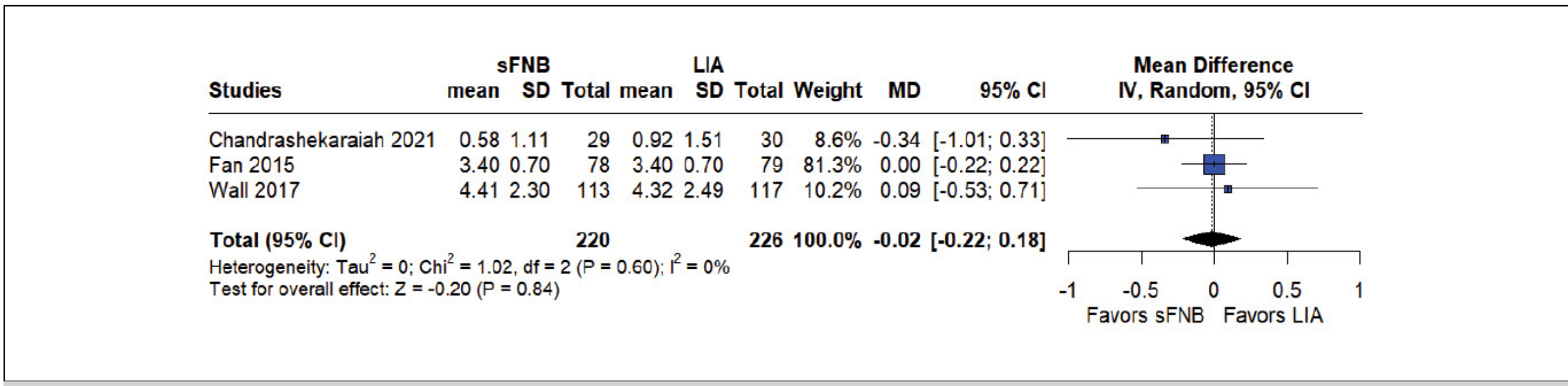

Supplementary Figure 7. Subgroup of studies that used morphine in the local infiltration analgesia cocktail for the outcome of pain scores on postoperative day 1

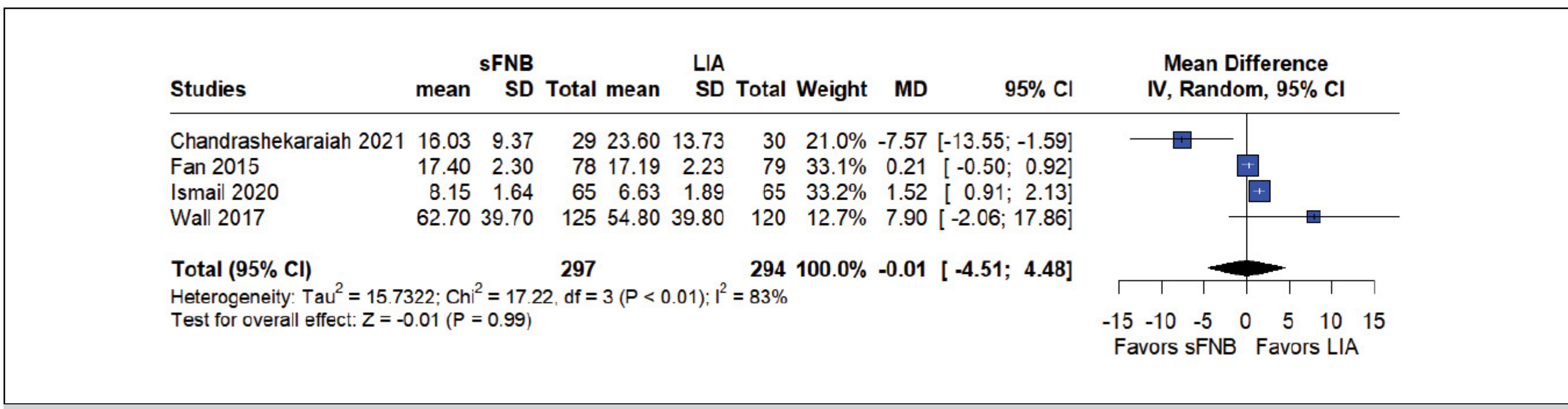

Supplementary Figure 8. Subgroup of studies that used morphine in the local infiltration analgesia cocktail for the outcome of opioid consumption on postoperative day 1.

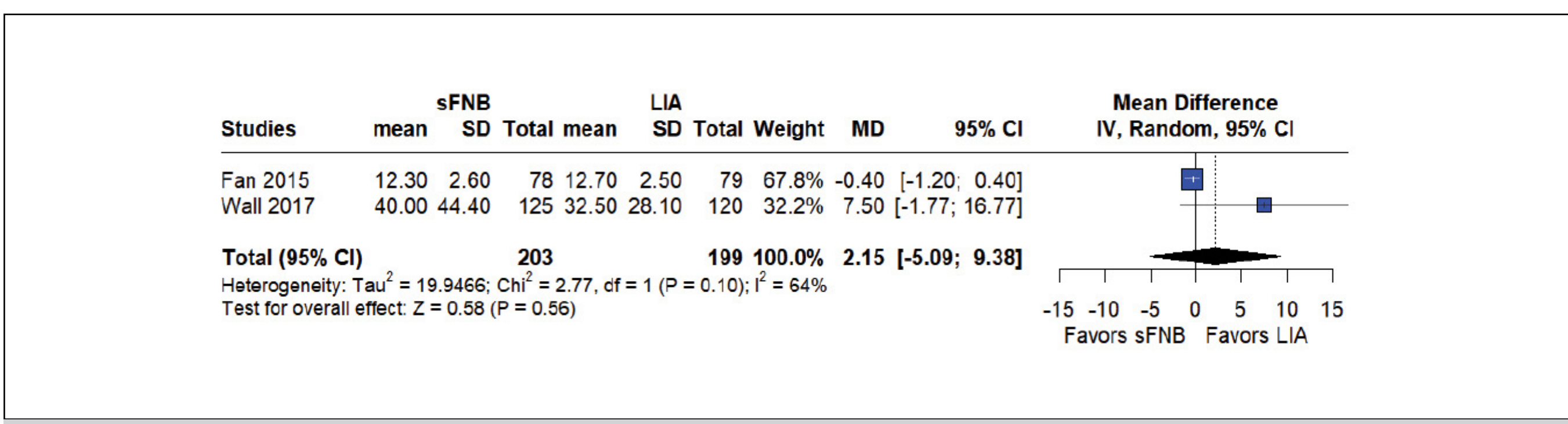

Supplementary Figure 9. Subgroup of studies that used morphine in the local infiltration analgesia cocktail for the outcome of opioid consumption on postoperative day 2.

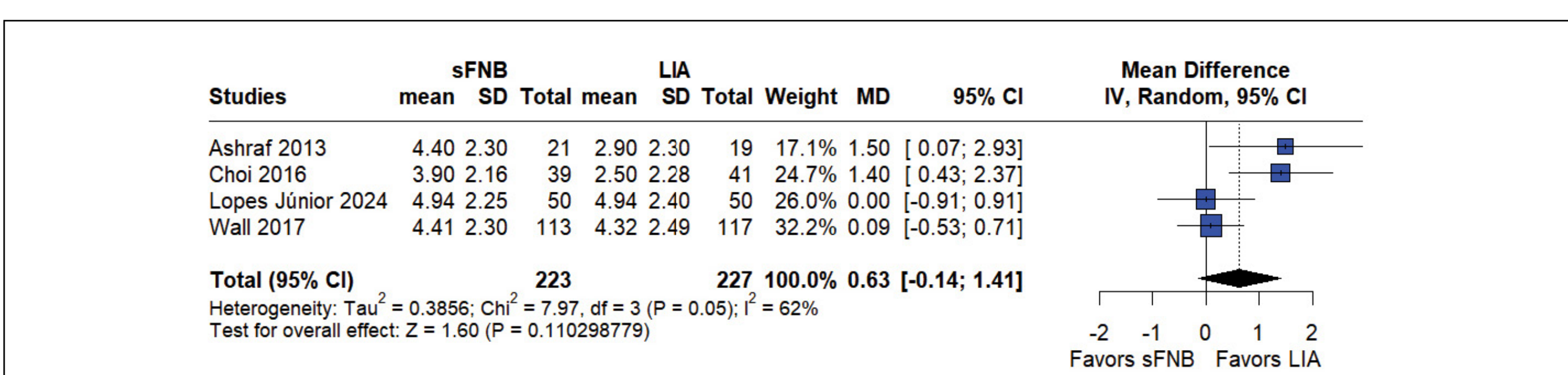

Supplementary Figure 10. Subgroup of studies that used ketorolac in the local infiltration analgesia cocktail for the outcome of pain scores on postoperative day 1.

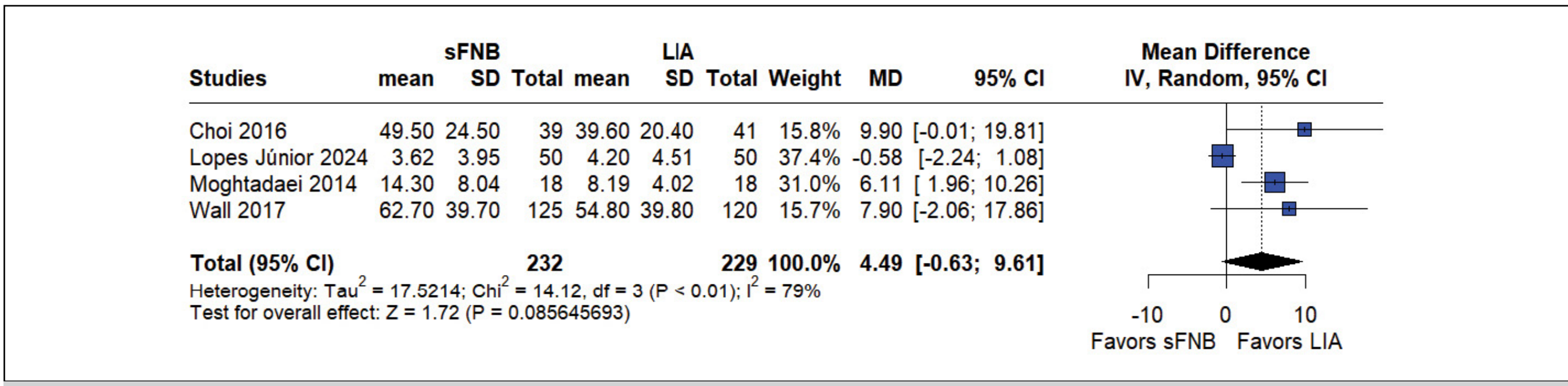

Supplementary Figure 11. Subgroup of studies that used ketorolac in the local infiltration analgesia cocktail for the outcome of opioid consumption on postoperative day 1.

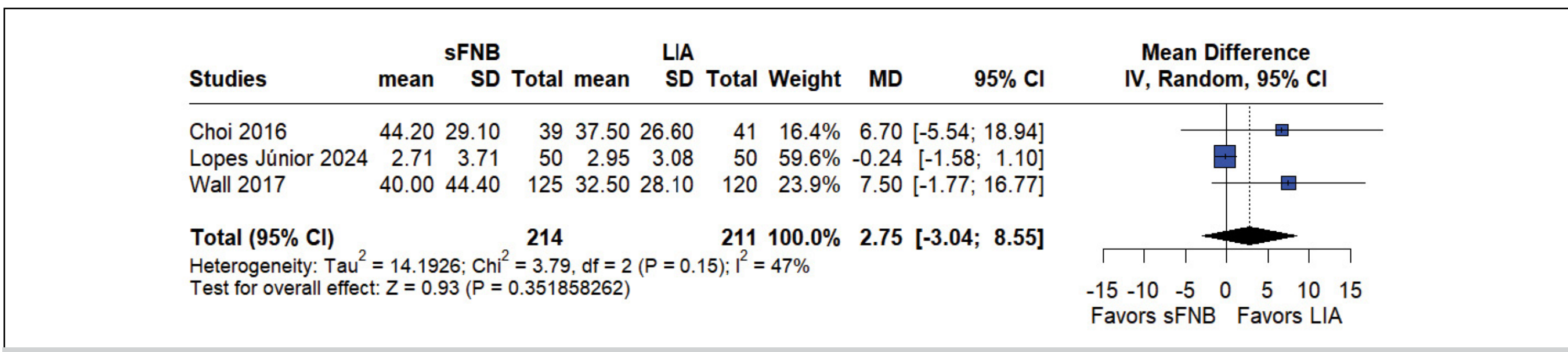

Supplementary Figure 12. Subgroup of studies that used ketorolac in the local infiltration analgesia cocktail for the outcome of opioid consumption on postoperative day 2.

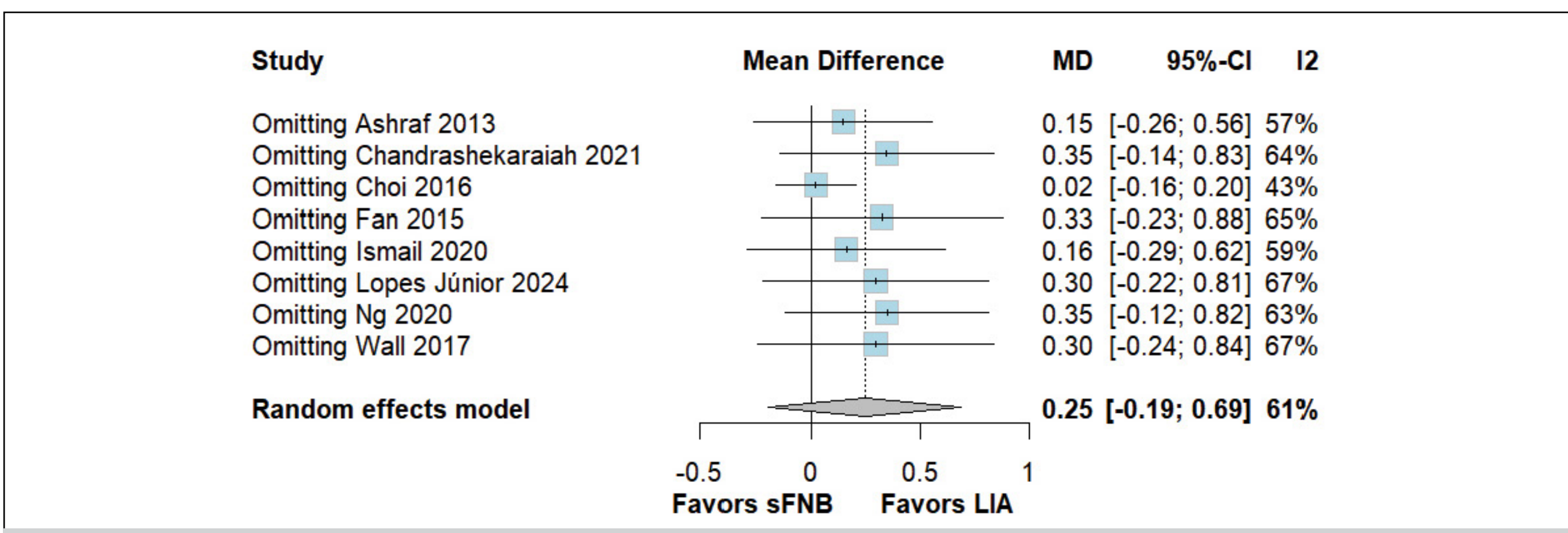

Supplementary Figure 13. Leave-one-out analysis of pain scores on postoperative day 1.

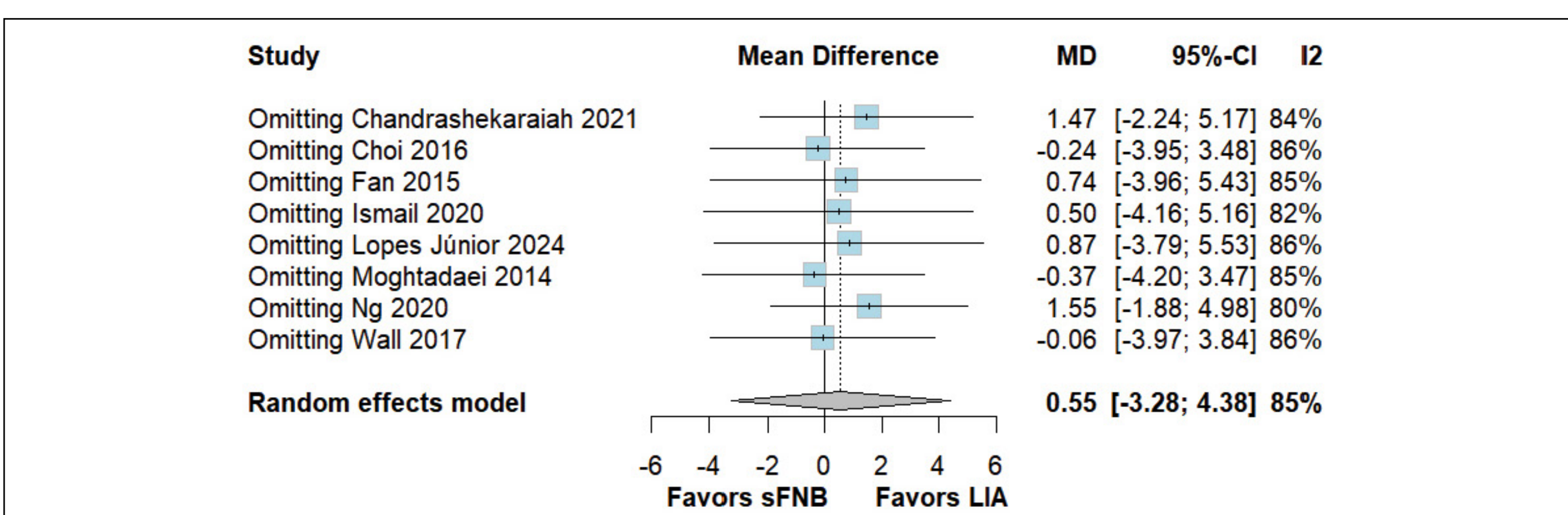

Supplementary Figure 14. Leave-one-out analysis of opioid consumption on postoperative day 1.

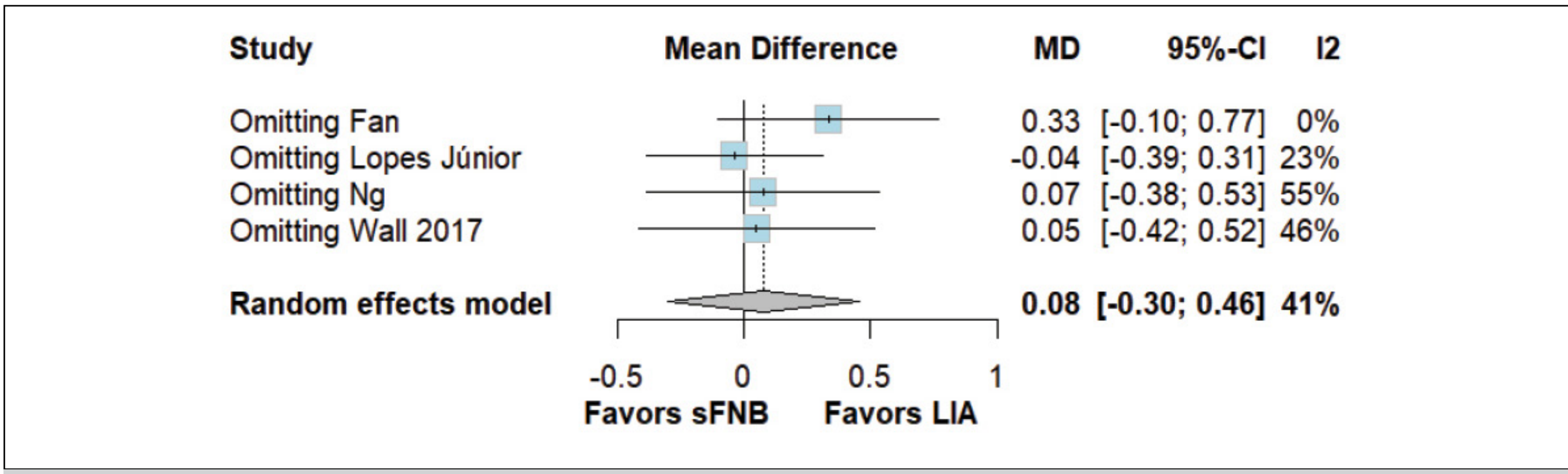

Supplementary Figure 15. Leave-one-out analysis of pain scores at rest on postoperative day 2.

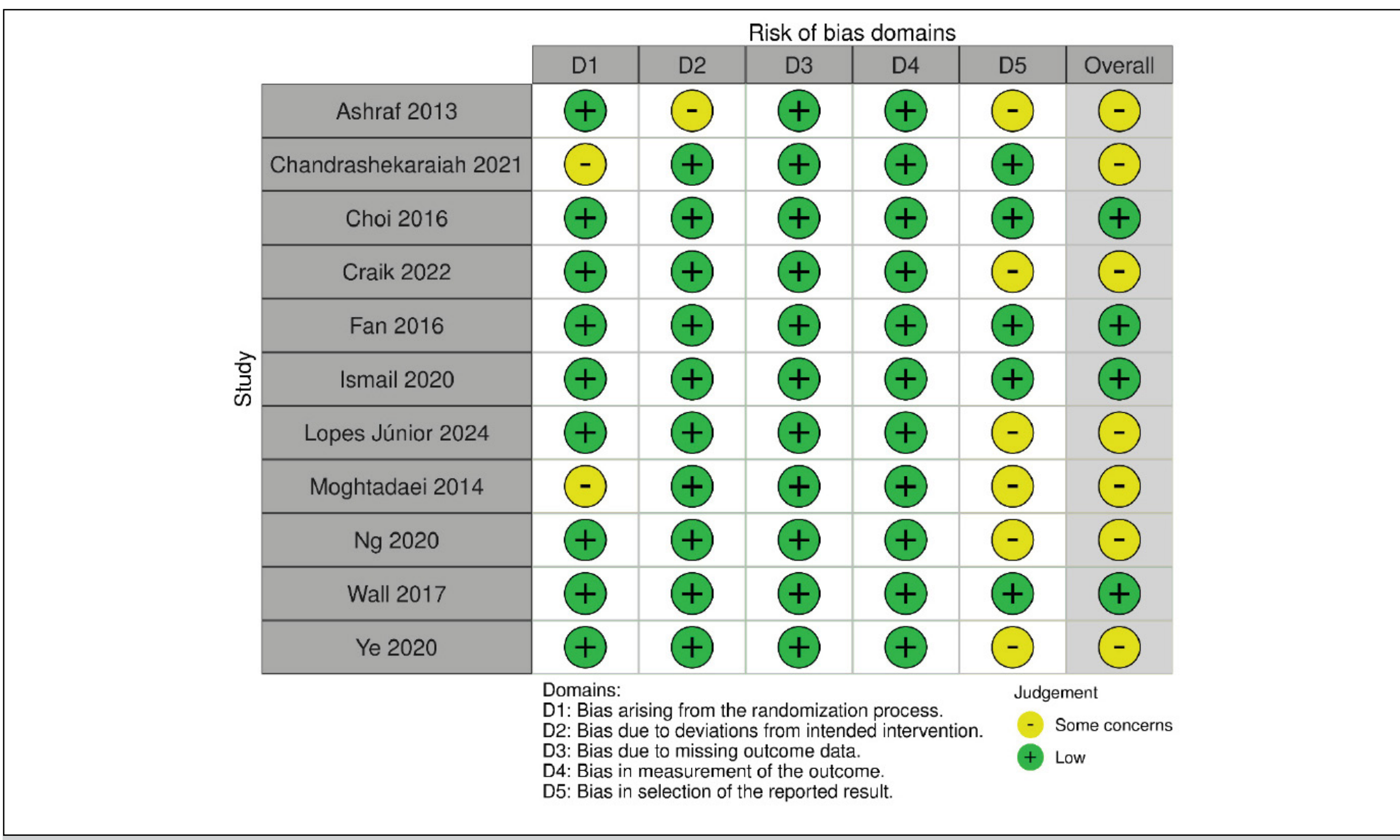

Supplementary Figure 16. Risk-of-bias assessment of the included studies using the Cochrane Risk of Bias 2 tool.

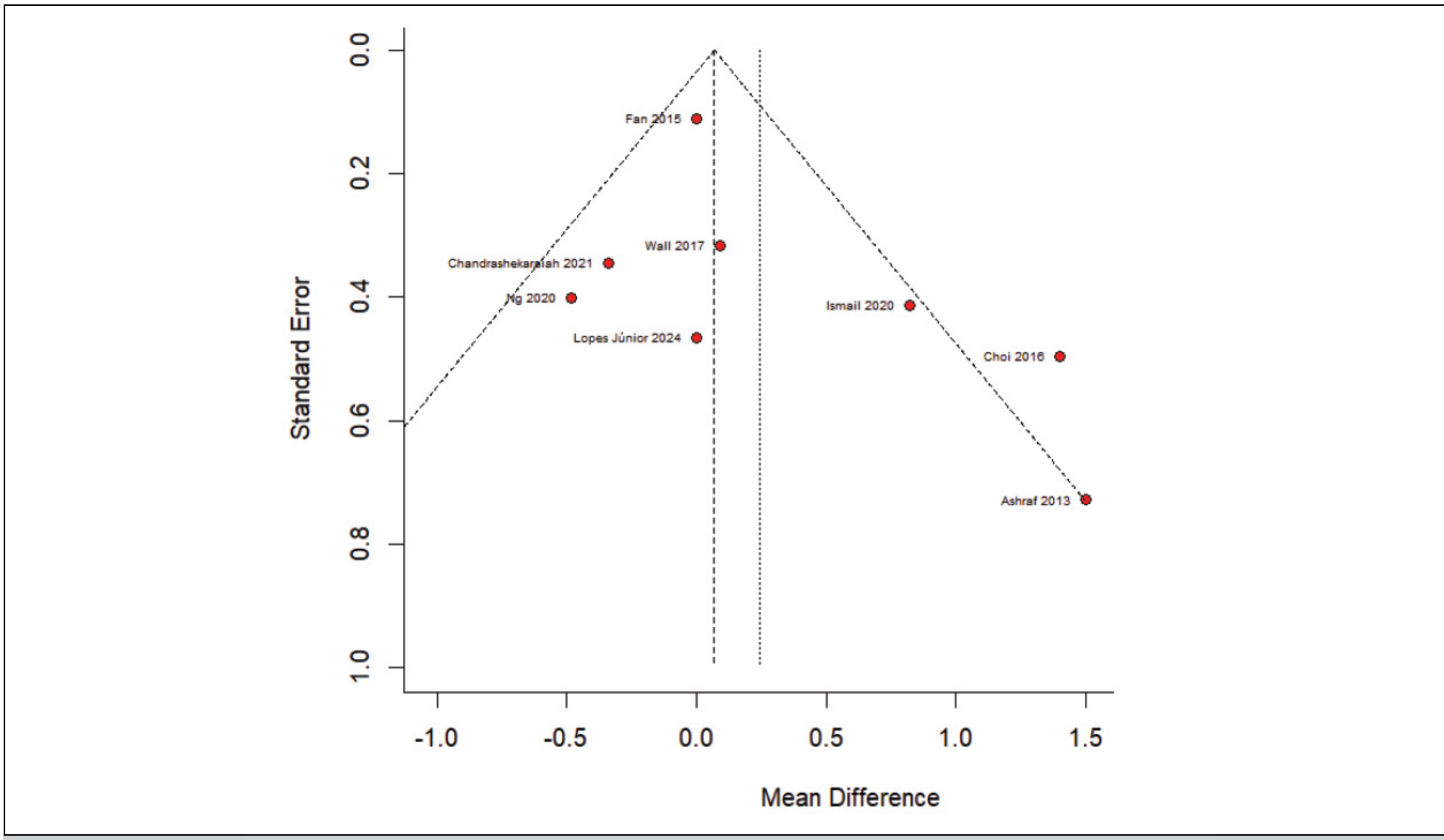

Supplementary Figure 17. Funnel plot of pain scores at rest on postoperative day 1.
